# Correction: Identifying social science engagement within agroecology: Classifying transdisciplinary literature with a semi-automated textual classification method

**DOI:** 10.1371/journal.pone.0315109

**Published:** 2024-12-03

**Authors:** Natalia Pinzón, Ryan E. Galt, Marcela Beatriz Baukloh Coronil

S1 Dataset is incorrect. Please view the correct S1 Dataset below.

## Supporting information

S1 Dataset Agroecology Corpus 2019: The results of the Agroecology Corpus with the SEAC method applied. (XLSX)
