# Supplementary material for: Identifying social science engagement within agroecology: Classifying transdisciplinary literature with a semi-automated textual classification method
Source: PLoS One. 2023 Feb 3;18(2):e0278991. doi: 10.1371/journal.pone.0278991 (PMC9897586; doi:10.1371/journal.pone.0278991)
Supplement: Attachment — Submitted filename: Pinzon_review.docx [file pone.0278991.s002.docx]

1. Is the manuscript technically sound, and do the data support the conclusions?
2. Has the statistical analysis been performed appropriately and rigorously?
3. Is the manuscript presented in an intelligible fashion and written in standard English?
4. Review Comments to the Author

I believe the method described in the paper is essentially sound. I am also glad that the method is being published in detail, as I suspect that much useful research methodology is more difficult to access and adopt than it needs to be.

As the point of the paper is to make a classification method accessible to transdisciplinary scholars, it’s important that it be presented as clearly as possible. While the manuscript has been carefully written overall, I do have some suggestions for improving the clarity and usability of the work:

1. I *highly recommend* adding a figure that gives an overview/conceptual model of the method. The first place to refer to the figure could be in the paragraph beginning at L141, where terms such as “multi-step filter” and “stepwise equation” might suggest to the reader that the process is more complex and technical than it really is. A figure could also help illuminate the iterative processes that are described in the Methods section.
2. The use of the word “citation” to describe the documents that were analyzed is somewhat confusing. On reading the abstract, I thought the MS would be about classifying documents based on cited references. Although ‘citations’ is technically correct, I would suggest either replacing it with a word like “documents”, “articles”, or “publications”, or at least, defining it very early in the text.
3. L32: “agroecology with undifferentiated engagement with social science (with 2,241 citations) or unclassifiable” – I didn’t realize until I’d summed up the numbers of citations that “unclassifiable” was a synonym for “agroecology with undifferentiated engagement”. Undifferentiated seems a bit jargon-y for a paper aimed at a wide variety of researchers. Why not just use unclassifiable instead?
4. L83: phenomenon 🡪 phenomena
5. L111: corpora 🡪 corpus
6. L332: It’s not clear what the ‘nine values’ are
7. L411: It would be good to state here that this is available in S2
8. L467: Did you assume that Agriculture and Human Values contained only social science papers (as implied here), or did you manually classify them (as stated on L487)?
9. L498: PESS or UESS? Please be consistent throughout.
10. L515: Here you contrast the “full” and “random” training sets. A table listing the training data sets and the number of ESS/NESS/UESS each one contained would be helpful.
11. L516 – end of paragraph: This is rather difficult to follow. I suggest rearranging the sentences so you start by explaining that the random dataset is the most representative so where it contains enough instances of a term (?), that dataset alone was used to estimate probabilities. If not, then…
12. L544 The text will read better if you start a new paragraph here. Also, you might as well round percentages to whole numbers throughout, your method isn’t accurate enough to justify stating those numbers to n decimal places.
13. L571: You were able to identify additional terms that appeared frequently in ESS but not in NESS citations and include them in the classification scheme. Doesn’t that mean that actually the expert consultation step isn’t strictly necessary? You could have written code to automate finding all of these terms. Do you feel that that would stray into ‘machine learning’, so the advantage of the expert consultation step simply that it involves less coding, perhaps making the method more accessible to a range of researchers? If so, do you want to acknowledge that either here or when you discussed the expert elicitation step? Are there other advantages to involving experts, such as comparing your findings about what classifies an agroecology text as ESS with their expectations?
14. L612 “We looked for patterns…” Similar to previous comment, you didn’t use a formal minimization/optimization/fitting procedure here. Why is that?
15. L692: Table 5?
16. L693: Applied by you to this dataset?
17. L831: It’s a factor of 3-4, not an order of magnitude
18. L895 specificity 🡪 sensitivity?
19. Will the Python script/associated materials contain instructions or a runnable example for reproducing the work done in the paper? If so, it would be good to state that explicitly, to encourage people to give it a try.
20. The title would more accurately read “*semi*-automated textual classification’

Also, here are a couple of suggestions for future work (not necessarily for this paper):

1. L888 mentions that the social science engagement in agroecology may change over time, and describes this as a weakness of the method. This actually seems like an interesting avenue for future work – although the list of social science terms may need to be re-created, that exercise could in itself reveal which theories, perspectives, lines of argument, etc. were important at different times in the development of the field.
2. It would be interesting to compare/corroborate the SEAC results using a text-based cluster analysis. Do papers classified as ESS and NESS form distinct clusters, with UESS scattered in between?
3. The authors may be interested in the open-access S2ORC corpus for future work: https://allenai.org/data/s2orc
