# Supplementary material for: Identifying social science engagement within agroecology: Classifying transdisciplinary literature with a semi-automated textual classification method
Source: PLoS One. 2023 Feb 3;18(2):e0278991. doi: 10.1371/journal.pone.0278991 (PMC9897586; doi:10.1371/journal.pone.0278991)
Supplement: Attachment — Submitted filename: Response to Reviewers.docx [file pone.0278991.s003.docx]

Dear Dr. Mason,

Thank you for your thoughtful and detailed comments. We are grateful for the level of attentiveness you gave to our manuscript. We have incorporated your suggestions into the text and have addressed your concerns below (responses in blue).

1. I *highly recommend* adding a figure that gives an overview/conceptual model of the method. The first place to refer to the figure could be in the paragraph beginning at L141, where terms such as “multi-step filter” and “stepwise equation” might suggest to the reader that the process is more complex and technical than it really is. A figure could also help illuminate the iterative processes that are described in the Methods section.
   a. This is an excellent suggestion. We have added Figure 1 to show an overview of the SEAC method in the intro. We also added Figure 2 to visually show the development of the SEAC method in the methods section.
2. The use of the word “citation” to describe the documents that were analyzed is somewhat confusing. On reading the abstract, I thought the MS would be about classifying documents based on cited references. Although ‘citations’ is technically correct, I would suggest either replacing it with a word like “documents”, “articles”, or “publications”, or at least, defining it very early in the text.
   a. We replaced “citations” with “publications” throughout
3. L32: “agroecology with undifferentiated engagement with social science (with 2,241 citations) or unclassifiable” – I didn’t realize until I’d summed up the numbers of citations that “unclassifiable” was a synonym for “agroecology with undifferentiated engagement”. Undifferentiated seems a bit jargon-y for a paper aimed at a wide variety of researchers. Why not just use unclassifiable instead?
   a. We changed “undifferentiated” to “unknown”. We chose to keep the acronym “UESS” (unknown engagement with social science) to stay consistent with “ESS” and “NESS”
4. L83: phenomenon > phenomena
   a. done
5. L111: corpora > corpus
   a. thank you, done
6. L332: It’s not clear what the ‘nine values’ are
   a. These are the nine values used in the Stepwise equation (Table 5). Since we don’t introduce the Stepwise equation until much later, and this paragraph is meant to be an overview of the method, we removed reference to the ‘nine values’. It now reads *“We then developed a stepwise equation to find thresholds that would allow us to determine whether a citation was engaged with social science (ESS) or not (NESS)” (Line 319)*
7. L411: It would be good to state here that this is available in S2
   a. Done.
8. L467: Did you assume that Agriculture and Human Values contained only social science papers (as implied here), or did you manually classify them (as stated on L487)?
   a. Good catch. We manually classified all training sets, including Agriculture and Human Values. We clarified this in the text in line 449
9. L498: PESS or UESS? Please be consistent throughout.
   a. PESS was used during manual classification only, UESS was one of the categories resulting from the SEAC method. Please see Line 477 for clarification
10. L515: Here you contrast the “full” and “random” training sets. A table listing the training data sets and the number of ESS/NESS/UESS each one contained would be helpful.
    a. We added Table 3 listing the training sets as requested
11. L516 – end of paragraph: This is rather difficult to follow. I suggest rearranging the sentences so you start by explaining that the random dataset is the most representative so where it contains enough instances of a term (?), that dataset alone was used to estimate probabilities. If not, then…
    a. Thank you, this paragraph has been rearranged.
12. L544 The text will read better if you start a new paragraph here. Also, you might as well round percentages to whole numbers throughout, your method isn’t accurate enough to justify stating those numbers to n decimal places.
    a. We accepted your suggestion to round to whole numbers, however we chose to keep one decimal point in Table 6 describing the performance of SEAC method
13. L571: You were able to identify additional terms that appeared frequently in ESS but not in NESS citations and include them in the classification scheme. Doesn’t that mean that actually the expert consultation step isn’t strictly necessary? You could have written code to automate finding all of these terms. Do you feel that that would stray into ‘machine learning’, so the advantage of the expert consultation step simply that it involves less coding, perhaps making the method more accessible to a range of researchers? If so, do you want to acknowledge that either here or when you discussed the expert elicitation step? Are there other advantages to involving experts, such as comparing your findings about what classifies an agroecology text as ESS with their expectations?
    a. There were some terms we sourced from experts which turned out to be useful in distinguishing between Engaged and Not Engaged in Social Science, but which we would not have found using our term likelihood script because they were so rare ( the script was designed to focus on terms with higher frequencies). Then there were some terms sourced only from the script and yet others suggested by experts which turned out not to be helpful. Overall, we believe the expert consultation step speeds up and improves the accuracy of the process significantly.
14. L612 “We looked for patterns…” Similar to previous comment, you didn’t use a formal minimization/optimization/fitting procedure here. Why is that?\
a. We clarified this in the text Line 592. Although we did not use a formal optimization method for this analysis because we opted for a less mathematically intensive approach, we would welcome others attempting to replicate our methods to use statistical optimization techniques if they find them to be an improvement on our method developed here.
15. L692: Table 5?
    a. Yes, thank you.
16. L693: Applied by you to this dataset?
    a. No these where separate studies with separate datasets. We edited the language for clarification.
17. L831: It’s a factor of 3-4, not an order of magnitude
    a. Yes, thank you
18. L895 specificity > sensitivity?
    a. Now L860 : The sentence was updated for clarity. Specificity was removed since we did not examine specificity in this study (though it was examined in the study referenced)
19. Will the Python script/associated materials contain instructions or a runnable example for reproducing the work done in the paper? If so, it would be good to state that explicitly, to encourage people to give it a try.
    a. Yes, good point. We have added a more explicit note in the last paragraph
20. The title would more accurately read “*semi*-automated textual classification’
    a. Agreed

Also, here are a couple of suggestions for future work (not necessarily for this paper):

Thank you for these excellent suggestions.

1. L888 mentions that the social science engagement in agroecology may change over time, and describes this as a weakness of the method. This actually seems like an interesting avenue for future work – although the list of social science terms may need to be re-created, that exercise could in itself reveal which theories, perspectives, lines of argument, etc. were important at different times in the development of the field.
   a. Good point, we have added this idea for others to consider
2. It would be interesting to compare/corroborate the SEAC results using a text-based cluster analysis. Do papers classified as ESS and NESS form distinct clusters, with UESS scattered in between?
   a. This is an excellent idea which we might consider implementing in future
3. The authors may be interested in the open-access S2ORC corpus for future work: <https://allenai.org/data/s2orc>
   a. Thank you for this resource!
